# DACH1: Its Role as a Classifier of Long Term Good Prognosis in Luminal Breast Cancer

**DOI:** 10.1371/journal.pone.0084428

**Published:** 2014-01-02

**Authors:** Desmond G. Powe, Gopal Krishna R. Dhondalay, Christophe Lemetre, Tony Allen, Hany O. Habashy, Ian O. Ellis, Robert Rees, Graham R. Ball

**Affiliations:** 1 The John van Geest Cancer Research Centre, Nottingham Trent University, Nottingham, United Kingdom; 2 Department of Cellular Pathology, Nottingham University Hospitals NHS Trust, Nottingham, United Kingdom; 3 Albert Einstein College of Medicine, Bronx, New York, United States of America; 4 Department of Computing and Informatics, Nottingham Trent University, Nottingham, United Kingdom; 5 Pathology Department, Faculty of Medicine, Mansoura University, Mansoura City, Daqahlia, Egypt; Wayne State University, United States of America

## Abstract

**Background:**

Oestrogen receptor (ER) positive (luminal) tumours account for the largest proportion of females with breast cancer. Theirs is a heterogeneous disease presenting clinical challenges in managing their treatment. Three main biological luminal groups have been identified but clinically these can be distilled into two prognostic groups in which Luminal A are accorded good prognosis and Luminal B correlate with poor prognosis. Further biomarkers are needed to attain classification consensus. Machine learning approaches like Artificial Neural Networks (ANNs) have been used for classification and identification of biomarkers in breast cancer using high throughput data. In this study, we have used an artificial neural network (ANN) approach to identify DACH1 as a candidate luminal marker and its role in predicting clinical outcome in breast cancer is assessed.

**Materials and methods:**

A reiterative ANN approach incorporating a network inferencing algorithm was used to identify ER-associated biomarkers in a publically available cDNA microarray dataset. DACH1 was identified in having a strong influence on ER associated markers and a positive association with ER. Its clinical relevance in predicting breast cancer specific survival was investigated by statistically assessing protein expression levels after immunohistochemistry in a series of unselected breast cancers, formatted as a tissue microarray.

**Results:**

Strong nuclear DACH1 staining is more prevalent in tubular and lobular breast cancer. Its expression correlated with ER-alpha positive tumours expressing PgR, epithelial cytokeratins (CK)18/19 and ‘luminal-like’ markers of good prognosis including FOXA1 and RERG (p<0.05). DACH1 is increased in patients showing longer cancer specific survival and disease free interval and reduced metastasis formation (p<0.001). Nuclear DACH1 showed a negative association with markers of aggressive growth and poor prognosis.

**Conclusion:**

Nuclear DACH1 expression appears to be a Luminal A biomarker predictive of good prognosis, but is not independent of clinical stage, tumour size, NPI status or systemic therapy.

## Introduction

Breast cancer is the most common cancer in females and the third most common cause of cancer death in the UK after lung and large bowel cancer [1]. Recent studies have confirmed the heterogeneity of breast cancer arising from inherited and acquired genetic variation. It has recently been proposed that 10 molecular breast cancer groups exist [2], building on the overarching and simpler four group molecular stratification established more than a decade ago [3]–[6]. The largest of these groups comprise oestrogen receptor (ER) positive (luminal) tumours with the latest evidence suggesting complex clinical diversity and mortality risk [2]. It has long been appreciated that the oestrogen receptor has a compelling role in breast cancer biology because its expression is both a predictive and independent prognostic factor for disease outcome, treatment response and recurrence in breast cancer [7]. This is because when activated it induces pro-cancerous cell signalling pathways, influencing cell growth, survival and differentiation.

Gene expression array data has shown the luminal family of breast cancer includes at least one high risk subgroup, several intermediate risk subgroups (including a luminal B subgroup), and two good prognosis subgroups comprising a ‘pure’ ER luminal A subgroup and a mixed ER positive/negative subgroup [2]. Improved classification delivering clinical utility is required to achieve more effective therapeutic treatment and to identify patients that will be refractory to anti-hormonal therapy. Luminal A tumours tend to be low grade tumours that are characterised by over expression of ER-activating genes including LIV1, CCND1, FOXA1, XBP1, GATA3 and Bcl-2 [8]. Contrasting with this, luminal B cancers are high grade, show increased proliferation (Ki67 positive) and growth factor receptors such as EGFR, and have variable HER2 expression [9]. A number of studies have attempted to phenotype luminal subgroups using protein biomarkers with immunohistochemistry, and to relate these to increased risk of adverse events. For example the transferrin receptor, CD71, is involved in the uptake of iron and is expressed on cells showing high proliferation, and previously we reported it to be an independent prognosticator of an ER+ subgroup characterised by poor prognosis and resistance to endocrine therapy [10]. Another example is the proliferation related marker TK1 which is an enzyme involved in the synthesis of thymidine triphosphate needed by the proliferating cells to enter S phase [11]. In addition, CARM 1 [12] and PELP1 are transcriptional corepressors and indicators of reduced disease free survival in luminal cancers [13]. PELP1 is a coactivator that binds with the AF-2 domain (oestrogen responsive element) of ERα, facilitating downstream estradiol-induced DNA synthesis and cell proliferation [14].

In recent times, various computational approaches have been developed for cancer classification and diagnosis prediction [15]. In breast cancer hierarchical clustering analysis of gene expression array data has proven useful in providing broad molecular classification [3], but other techniques are required to identify biomarkers defining membership to various subgroups. Subsequently, computer algorithms incorporating a multilayer perceptron based Artificial Neural Network (ANN) method [16] have been adopted to identify cancer-relevant biomarkers to assist in clinical decision-making [17], [18]. Previously ANN has been used to identify a panel of protein biomarkers [19] capable of classifying breast cancer patients parallel to that achieved using gene expression profiling [3]. ANNs have proved to be capable of modelling biological systems more precisely than conventional statistical techniques [20], and are successful for avoiding over-fitting and to produce generalised models with validation subsets in breast cancer dataset [21].

In this study we used an ANN based network inference approach [22] to identify ER-associated biomarkers with the aim of improving classification of luminal breast cancer group based on cancer specific survival. Seventeen candidate genes were identified including the Drosophila dachshund (dac) gene. DACH1 belongs to the nuclear protein family undertaking a vital role in promoting differentiation of Drosophila eye and limb and retinal determination signalling pathway [23], [24]. In humans, DACH1 is known to repress tumorigenesis in human breast and prostate cancers [25] and down regulates EGFR and cyclin D1 in tumour cells [26]. Furthermore, DACH1 may control stem cell gene expression [27] preventing cancer cell migration needed for metastasis development [28]. DACH1 was selected for further study because it is hypothesised that high levels of DACH1 will competitively inhibit the growth promoting activity of PELP1 and consequently will be associated with improved prognosis. The current study aims to characterise the association of DACH1 with other cancer relevant biomarkers in the luminal subtype of breast cancer, with the emphasis being in determining its possible role as a clinical classifier of disease outcome and as a prognostic biomarker.

## Materials and Methods

This study was approved by the Nottingham Research Ethics Committee 2 under the title ‘Development of a molecular genetics classification of breast cancer’.

### Breast cancer microarray dataset

To identify genes associated with ER status in breast cancer a cDNA microarray dataset, E-GEOD-20194 [29], was selected from the public repository ArrayExpress [30], submitted by Micro Array Quality Control consortium. The dataset comprises expression values for 22,283 probe sets targeting gene transcripts across 278 samples (ER positive = 164 and ER negative = 114) with tumour stage ranging from I-III.

### ANN architecture and model development

The ANN architecture encompasses supervised learning from a multilayer perceptron model employing two hidden nodes with a sigmoidal transfer function. The samples were subjected to Monte Carlo Cross Validation strategy by randomly segregating them into three different subsets namely: train (to perform learning), test (for early stopping when the network fails to perform better with a threshold of 3000 epochs or 1000 epochs without improvement in mean square errors (MSE) and validation subsets (to authenticate the model performance on previously unseen data) in a proportion of 60%, 20% and 20% respectively. Each of the 22,283 probe sets were used as individual input variables in the model. The algorithm used a momentum of 0.5 and learning rate of 0.1. The error differences in actual and predicted values were used to update the weights with a back propagation algorithm. The complete ANN model is reiterated 50 times with random sampling. Across 50 ANN model predictions, the average MSE of a test subset for each input variable was considered to determine their predictive capability for ER class.

### Interaction network development

To evaluate the interactions between the highly predictive probe sets for ER class, we have employed the interaction algorithm based on an ANN model described earlier [31]. Briefly, from a set of 100 probes, 99 probe expression values (inputs) were used to predict a single one (output). An ANN model was trained until an optimal solution is found minimising the difference between the expected output and the predicted. The weights for the optimised model were recorded. This process was iteratively repeated, selecting new inputs from the 100 set, until all probe expressions are predicted from the remaining probes. The weights quantify the intensity of the relation between source and target which could be positive (stimulating) or negative (inhibiting). The analysis generated a matrix of 9,900 bidirectional interactions for all 100 probes. These were subsequently filtered to select the top 100 interactions for further visualisation.

The interaction network was visualised using Cytoscape® Ver 2.7.0 [32], which symbolised each probe set as a node and interaction as an edge. To give directionality for the interactions each input was considered as source, the output as target, and the weights recorded for the prediction as interaction values. The directionality for the edge is given according to source and target, and the weight of the interaction is materialised by the thickness of the edges.

### Patient selection and immunohistochemistry

Tissue microarray (TMA) sections comprising 993 patients from the Nottingham Tenovus study (1986–1998) with two tissue cores represented from each patients tumour. TMA sections were immunostained to assess the protein expression levels of DACH1. This TMA is well characterised with data for clinical information, tissue protein expression of tumour-relevant pathological biomarkers and long term clinical follow-up including information on local, regional and distant tumour recurrence, and cancer specific survival outcome [10]. Patient management was based on the Nottingham Prognostic Index (NPI) score and ER status as previously described [33]. Breast cancer specific survival (BCSS) was defined as the time (in months) from the date of the primary surgical treatment to the time of death from breast cancer. Distant metastasis free interval (DMFI) was defined as the interval (in months) from the date of the primary surgical treatment to the date of development of the first distant metastasis.

Four micron thick formalin fixed paraffin-processed TMA and full face sections were subjected to microwave antigen retrieval in citrate buffer (pH 6.0), and then immunohistochemically stained with a rabbit polyclonal antibody against DACH1 (Sigma HPA012672, St Louis, USA) using a streptavidin biotin technique (Dako, Cambridge, UK). The DACH1 antibody was optimised for heterogeneity and specificity at a working dilution of 1∶200. Sections were counterstained in haematoxylin and mounted using DPX mounting medium. Negative controls comprising omission of the primary antibody or substitution with an inappropriate primary antibody of similar immunoglobulin class was used.

The immunohistochemically stained TMA sections were scored with observers blinded to the clinicopathological features of tumours and patients' outcome. Nuclear staining intensity and percentage of cells stained was assessed in unequivocal malignant epithelium using the H-score (histochemical score) [34]. Staining intensity was scored 0, 1, 2 or 3 and the percentage of positive cells at each intensity subjectively estimated to produce a final score in the range 0–300. Damaged tissue cores and those that did not contain invasive carcinoma were censored.

### Statistical Analysis

Statistical analysis was performed using SPSS 15.0 (SPSS Inc., Chicago, IL, USA) software. Three patient subgroups were identified representing negative, low and high tumour nuclear H-scores. The Kaplan-Meier method with a log rank test was used to model the association of DACH1 group membership with cancer specific survival. Patients were categorised using an H-score ≥200 to define strong DACH1 positivity obtained in the majority of cells in a patient's tumour. Association between DACH1 expression and different clinicopathological factors and breast cancer markers was evaluated using the non-parametric Chi-square test. Patients that died due to causes other than breast cancer were censored during survival analyses. Multivariate Cox proportional hazard regression models were used to evaluate any independent prognostic effect of the variables with 95% confidence interval. A *p*-value of <0.05 was considered to indicate statistical significance.

## Results

### Identification of the ER interactome

Details of the gene signature associated with ER status were recently published[22]. The best predictive probe sets for showing association with ER status were selected based on lowest average of test error encountered across 10 independent predictive models. The best predictive probe was found to be 205225_at belonging to ESR1 gene which codes for oestrogen receptor alpha (ERα). Other highly predictive probe sets included GATA3, CA12 and NAT1 and DACH1 (205471_s_at).

### Interaction network inference

The 100 best ER predictive probes selected from ER-positive samples were further submitted to a network inference algorithm to determine the strength and nature of interactions between the selected probes. The algorithm yielded 9,702 interactions across 10 independent models. To reduce the dimensionality and to remove insignificant interactions, a filtering strategy was applied to select only the top 200 interactions based on interaction weight. Bidirectional interactions were computed for any given pair of genes accordingly to yield a bidirectional interaction matrix between each source and target.

A network model of the top 200 (100 positive and 100 negative) interactions forming positive and negative hubs is shown in Figure S1. For example, DACH1 (Dachshund homolog 1), SERPINA 5 (Serpin peptidase inhibitor member 5), TFF3 (Trefoil factor 3), and RARA (Retinoic acid receptor alpha) were connected with the majority of positive interactions forming positive hubs. In contrast, SOX11 (SRY (sex determining region Y)-box11), EGFR (Epidermal growth factor receptor) and CDH3 (cadherin 3, type 1, P-cadherin) were connected with the majority of negative interactions forming negative hubs. The strongest positive influence was found between TFF1 (Trefoil factor 1) and TFF3, and the strongest negative influence was found between MAPT (Microtubule-associated protein tau) and EGFR.

**Figure 1:**
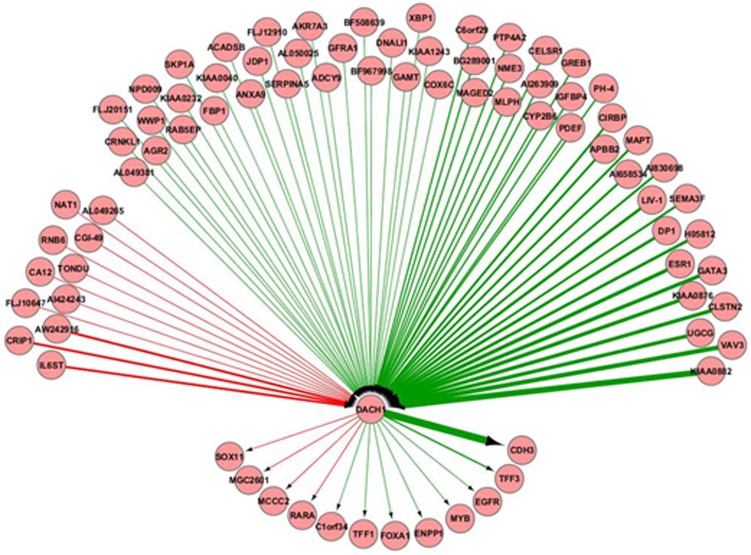
DACH1 Interactome. The association of DACH1 with the 100 best predictive genes in ER-positive tumours. The genes are represented as nodes and interactions as edges. The green edge is a positive interaction and the red edge is a negative interaction. The intensity of the interaction is represented in terms of the thickness of edge and the directionality with the arrow.

To establish an interaction map with only DACH1 in luminal (ER-positive) breast cancer samples, we created a DACH1 interactome (Figure 1) using the 100 best predictive genes. Computationally, DACH1 was found to be highly positively influenced by KIAA0882, a variant of TBC1 (tre-2/USP6, BUB2, cdc16 domain 1) family member 9A, and highly negatively influenced by IL6ST (Interleukin 6 signal transducer). DACH1 was also found to be highly positively and negatively influencing CDH3 and SOX11 respectively. An interaction map (Figure S2) of important genes overlapping with the oestrogen receptor and DACH1 respectively, shows similarity.

### DACH1 protein expression in breast cancer

**Figure 2:**
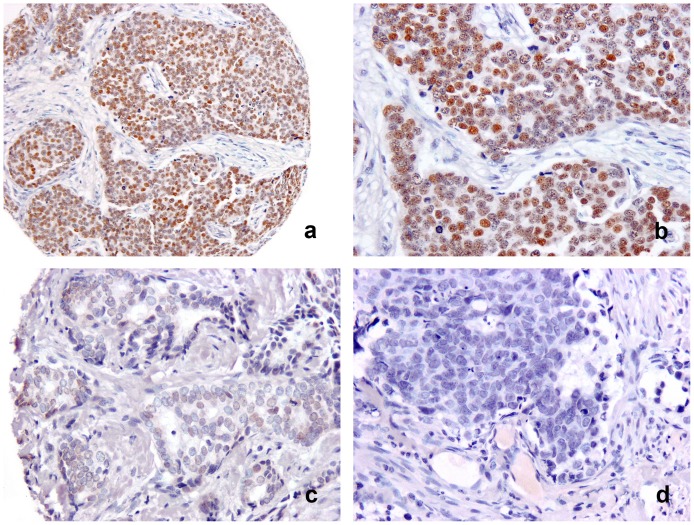
Nuclear DACH1 immunostaining varied in intensity from being strong with expression in a high proportion of cells (a, b), to weak (c) or negative (d), in breast carcinoma.

To test the clinical relevance in breast cancer, the association of DACH1 protein with clinicopathology features was investigated in a well characterised patient cohort. The median age of the patients was 55 years (range 27–70). DACH1 immunostaining was localised to the nuclei of malignant cells and was found to be either negative, weak or strong in intensity (Figure 2). DACH1 was significantly increased in post-menopausal patients with lobular and tubular cancer types but in contrast, was rarely seen in patients with medullary cancer. DACH1 expression showed no significant association with tumour size, tumour stage, metastasis development, tumour recurrence, or vascular invasion. DACH1 expression was significantly increased in tumours of low grade, good Nottingham Prognostic Index and candidacy for hormonal therapy (Table 1).

**Table 1:** Association of DACH1 expression with clinicopathological factors. N = number of samples. Statistically significant p-values are in bold.

Clinical Parameter	DACH1 absent		DACH1 present		Chi-square (X^2^)	p-value
	N	%	N	%		
**Age group**					12.505	**0.006**
<40	40	10.31	35	5.79		
40–50	128	32.99	176	29.09		
51–60	124	31.96	197	32.56		
60–75	96	24.74	197	32.56		
**Menopause**					8.912	**0.003**
Premenopausal	174	44.85	214	35.37		
Postmenopausal	214	55.15	391	64.63		
**Tumour Size**					2.283	0.131
≤1.5 cm	178	46.23	307	51.17		
>1.5 cm	207	53.77	293	48.83		
**Tumour Stage**					0.413	0.813
1	241	62.27	362	60.23		
2	112	28.94	183	30.45		
3	34	8.79	56	9.32		
**Tumour Grade**					69.335	**<0.001**
1	35	9.09	134	22.33		
2	94	24.42	226	37.67		
3	256	66.49	240	40.00		
**Nottingham Prognostic Index**					22.571	**<0.001**
Good	75	19.48	200	33.28		
Moderate	233	60.52	309	51.41		
Poor	77	20.00	92	15.31		
**Tumour type**					57.194	**<0.001**
Ductal - Non Specific Type (NST)	260	68.60	314	53.04		
Lobular (Classical and variants)	28	7.39	85	14.36		
Tubular & Tubular mixed	50	13.19	136	22.97		
Medullary	20	5.28	3	0.51		
Special type (Mucinous, Cribriform and Invasive papillary)	4	1.06	14	4.36		
Mixed NST with Lobular and special types	17	4.49	40	6.76		
**Distant metastasis formation**					0.349	0.555
Absent	268	69.43	425	71.19		
Present	118	30.57	172	28.81		
**Tumour recurrence**					0.078	0.780
Absent	231	60.63	353	59.73		
Present	150	39.37	238	40.27		
**Vascular invasion**					5.345	0.069
Negative	222	57.81	325	54.53		
Probable	33	8.59	80	13.42		
Definite	129	33.59	191	32.05		
**Endocrine therapy received**					9.085	**0.003**
Untreated	261	71.12	331	61.41		
Treated	106	28.88	208	38.59		

### Association of DACH1 with disease biomarkers

Nuclear DACH1 expression was strongly increased in patients with ER-alpha positive tumours co-expressing PgR, and epithelial CK18/19 cytokeratins. Nuclear staining was significantly associated with ‘luminal-like’ markers of good prognosis including FOXA1 and RERG. In contrast, strong inverse associations were found with candidate luminal markers of poor prognosis including CD71 (Table 2).

**Table 2:** Association of DACH1 protein with other breast cancer biomarkers.

Markers		DACH1 absent		DACH1 present		Chi-square	
		N	%	N	%	(X^2^)	p-value
**ER**						142.867	**<0.001**
	Absent	181	49.45	78	13.66		
	Present	185	50.55	493	86.34		
**PgR**						55.671	**<0.001**
	Absent	212	58.56	191	33.69		
	Present	150	41.44	376	66.31		
**CK18**						54.282	**<0.001**
	Absent	86	24.86	39	7.21		
	Present	260	75.14	502	92.79		
**CK19**						5.786	**0.016**
	Absent	50	13.51	50	8.61		
	Present	320	86.49	531	91.39		
**HER2**						6.595	**0.010**
	Absent	311	83.38	524	89.12		
	Present	62	16.62	64	10.88		
**E-cadherin**						**0.853**	0.356
	Absent	145	40.06	213	37.04		
	Present	217	59.94	362	62.96		
**EGFR**						6.371	**0.012**
	Absent	249	76.62	425	83.66		
	Present	76	23.38	83	16.34		
**CK5/6**						66.158	**<0.001**
	Absent	267	71.97	534	91.75		
	Present	104	28.03	48	8.25		
**CK14**						11.671	**0.001**
	Absent	304	82.83	518	90.40		
	Present	63	17.17	55	9.60		
**p53**						33.999	**<0.001**
	Absent	227	62.71	457	80.04		
	Present	135	37.29	114	19.96		
**MIB1**						28.563	**<0.001**
	Absent	59	29.95	154	54.61		
	Present	138	70.05	128	45.39		
**FOXA1**						26.495	**<0.001**
	Absent	178	62.9	174	43.0		
	Present	105	37.1	231	57.0		
**CD71**						25.926	**<0.001**
	Absent	90	32.4	220	51.9		
	Present	188	67.6	204	48.1		
**PELP1**						0.375	0.540
	Absent	250	87.4	369	85.8		
	Present	36	12.6	61	14.2		
**RERG**						4.291	**0.038**
	Absent	214	78.7	306	71.7		
	Present	58	21.3	121	28.3		

Supporting its association with good prognosis, tumour DACH 1 expression correlated with low cell proliferation (MIB1). Low DACH1 frequency and expression was seen in tumours bearing markers of poor prognosis including the basal-like markers CK14/5/6 and EGFR, as well as HER2 and p53 positivity.

### Patients' outcome

**Figure 3:**
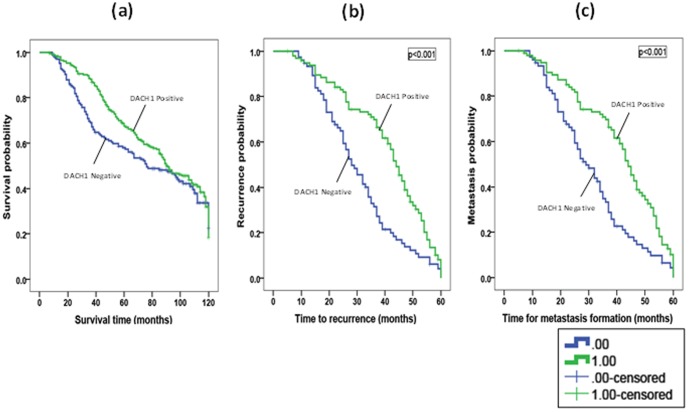
Kaplan-Meier plots modelling DACH1 expression with 5 year post-diagnostic a) specific survival, b) tumour recurrence, and c) distant metastasis. All were significant at p<0.001.

Patients with nuclear DACH1 positivity showed a significant association with cancer specific survival (n = 81 (54%); χ2 = 11.96, p<0.001), disease free interval (n = 81 (54%); χ2 = 15.33, p<0.001), tumour recurrence (n = 72 (52%); χ2 = 16.49, p<0.001) and distant metastasis (n = 72 (51%); χ2 = 16.31, p<0.001) over 5 years post diagnosis (Figure 3). However, the level of significance lessened for predicting cancer specific survival (χ2 = 2.31, p = 0.13), disease free interval (χ2 = 1.75, p = 0.17), tumour recurrence (χ2 = 2.11, p = 0.15) and distant metastasis (χ2 = 3.74, p = 0.053) over 10 years.

The effect of endocrine therapy on the ability of DACH1 to predict breast cancer specific survival was considered using Kaplan-Meier modelling. DACH1 positivity was associated with good survival in patients treated with tamoxifen (χ^2^ = 8.30, p = 0.004) and in addition, also showed a trend in patients not receiving tamoxifen (χ^2^ = 3.7, p = 0.055).

The predictive independence of DACH1 was tested using multivariate models (Cox regression) incorporating endocrine therapy, clinical stage, tumour size and NPI status. DACH1 was not found to be independent of these variables for predicting cancer specific survival.

## Discussion

In our study, we used an artificial neural network (ANN) based inference technique to identify ER associated biomarkers capable of separating good and poor prognosis patients with luminal type breast cancer. Consistent with expectations, the best predictive probe for identifying ER status in multiple independent runs was 205225_at representing ESR1 gene coding for oestrogen receptor alpha. Moreover the regulatory gene DACH1, associated with TGFβ signalling, was identified among the probe sets that produced a strongly positive interaction with ER status and so we tested its relevance as a luminal marker of disease progression by investigating its association with clinicopathologic variables. The objective is to compile cumulative evidence to produce a panel of markers capable of clinically guiding in the selection and management of breast cancer patients within the heterogeneous luminal class.

We observed three predominant patterns of nuclear DACH1 expression compatible with TSG (tumour suppressor gene) functionality. Nuclear DACH1 protein expression was significantly associated with markers of good prognosis including low cellular proliferation (MIB1 expression) and functional apoptosis (Bcl2 expression). It has previously been observed that reduced DACH1 expression occurs in invasive cancer compared to normal breast epithelium confirmed by our findings where DACH1 expression showed an inverse association with mitosis and cyclin D1 expression in breast cancer patient samples [26]. More recently, increased DACH1 expression was reported to correlate with reduced expression of IL-8 and other related chemokines, thus inhibiting cellular migration and invasion in MCF10A breast cancer cells [28]. Further evidence of its TSG function is provided by the observation that DACH1 homozygous deletion stimulates tumorigenesis in glioma cells [35], and loss of DACH1 occurs in high FIGO surgical stage endometrial cancers [36]. Furthermore, it has also recently been reported that over-expression of DACH1 protein is associated with poor prognosis when expressed in the cytoplasm rather than nuclei of ovarian cancer cells indicating disease progression [37], compatible with loss of TSG function. *In vitro* cell signalling studies have shown that DACH1 exerts its regulatory control on TGFβ signalling by nuclear binding via SMAD4 [26], [38], competing with precancerous transcriptional factors. Recent breast cancer studies have shown that DACH1 can directly influence the gene expression of stem cells, causing them to under-express CD24 [27]. In addition, it appears that the tumour suppressor function of DACH1 can be moderated by the tissue microenvironment including the presence of growth factors, evidenced by tumorigenesis seen in cell lines grown *in vitro* in the presence of IGF-1[39].

Steroid receptors, coactivators and co-repressors regulate the activity of ERα. PELP1 (proline, glutamic acid and leucine rich protein 1) is a coactivator that binds with the AF-2 domain (oestrogen responsive element) of ERα, facilitating downstream estradiol-induced DNA synthesis and cell proliferation [14]. Previously, we reported that PELP1 expression is associated with larger tumours and clinicopathology features indicative of poor prognosis, including high grade and basal cytokeratin expression [13]. DACH1 competitively binds with ERα, preventing PELP1 binding [14]. In the current study we found that moderate to high tumour nuclear DACH1 expression in the majority of cancer cells is compatible with functionally blocking PELP1 activity, reflected by its association with good prognosis. Conversely, absent or weak DACH1 nuclear staining represents unopposed PELP1 mediated tumour cell growth.

An inverse relationship was seen between DACH1 and basal type markers including CK14, CK5/6 and EGFR. EGFR is a member of the HER family associated with multiple downstream cell signalling pathways leading to adverse clinical outcomes including tumour growth and metastasis. In accord we found an inverse association for DACH1 and HER2. In this respect and similar to our previous report, we propose that DACH1 and FOXA1 [33] share membership of the Luminal A biomarker group in being associated with variables of good prognosis. DACH1 was found to be a predictor of specific survival but was not independent of hormonal therapy, clinical stage, tumour size or NPI status. Clinical tests that identify high risk (increased metastatic potential) patients with breast cancer to select candidates for chemotherapy treatment are currently under review [40]. Applying rationalised targeted treatment is necessary because chemotherapy can result in medical complications, reduced quality of life and economic burden. Crucially, some cancers present with no greater mortality risk if untreated with chemotherapy and among these, patients with Luminal A cancers appear to have good survival prospects (in press). Further investigation is required to determine if DACH1 and other Luminal A biomarkers can be used for selecting patients not requiring chemotherapy.

As ANNs have a proven application in breast cancer patient classification [22] and for biomarker identification associated with disease progression [41], in the current study the focus for relevance to clinical outcome has been exploited. Among the top ten ranked genes with positive association to ER was the transcription factor GATA3 known to be associated with ER [42], ER status [21] and hormonal responsiveness in breast cancer [43]. Genes showing a negative association with ER included CA12 which is associated with hypoxia and poor prognosis in breast cancer [44]. These findings and others in previous studies support the validity and robustness of the ANN technique and its application in identifying breast cancer biomarkers.

In summary, we have shown that DACH1 occurs in patients with ER+ breast cancers and predicts good prognosis. In this respect DACH1 can be regarded as a Luminal A biomarker.

## Supporting Information

Figure S1 **Interaction map of 2 (100 positive and 100 negative) interactions from highly predictive probe sets in ER positive samples.** The genes are represented as nodes and interactions as edges. Green edge is a positive interaction and red edge is a negative interaction. The intensity of the interaction is represented in terms of the thickness of edge and the directionality with the arrow from source to target. The nodes with multiple interactions (>5) are considered as hubs.
(TIF)

Figure S2 **Association of luminal markers with (a) ESR1 and (b) DACH1 in luminal samples.** The genes are represented as nodes and interactions as edges. The green edge is a positive interaction and the red edge is a negative interaction. The intensity of the interaction is represented in terms of the thickness of edge and the directionality with the arrow.
(TIF)

## References

[1] Cancer Research UK website. Available: http://publications.cancerresearchuk.org/downloads/Product/CS_KF_BREAST.pdf. Accessed 2012 Mar 21.

[2] Curtis C, Shah SP, Chin SF, Turashvili G, Rueda OM, et al (2012) The genomic and transcriptomic architecture of 2,000 breast tumours reveals novel subgroups. Nature 486: 346–352. 22522925 10.1038/nature10983 PMC3440846

[3] Perou CM, Sorlie T, Eisen MB, Van de Rijn M, Jeffrey SS, et al (2000) Molecular portraits of human breast cancr. Nature 406: 747–752. 10963602 10.1038/35021093

[4] Sorlie T, Perou CM, Tibshirani R, Aas T, Geisler S, et al (2001) Gene expression patterns of breast carcinomas distinguish tumor subclasses with clinical implications. Proc Natl Acad Sci U S A 98: 10869–10874. 11553815 10.1073/pnas.191367098 PMC58566

[5] Sorlie T, Tibshirani R, Parker J, Hastie T, Marron JS, et al (2003) Repeated observation of breast tumor subtypes in independent gene expression data sets. Proc Natl Acad Sci U S A 100: 8418–8423. 12829800 10.1073/pnas.0932692100 PMC166244

[6] Sotiriou C, Neo SY, McShane LM, Korn EL, Long PM, et al (2003) Breast cancer classification and prognosis based on gene expression profiles from a population-based study. Proc Natl Acad Sci U S A 100: 10393–10398. 12917485 10.1073/pnas.1732912100 PMC193572

[7] Harris L, Fritsche H, Mennel R, Norton L, Ravdin P, et al (2007) American Society of Clinical Oncology 2007 update of recommendations for the use of tumor markers in breast cancer. J Clin Oncol 25: 5287–5312. 17954709 10.1200/JCO.2007.14.2364

[8] Lin CY, Strom A, Vega VB, Kong SL, Yeo AL, et al. (2004) Discovery of estrogen receptor α target genes and response elements in breast tumor cells. Genome Biology5.. 10.1186/gb-2004-5-9-r66 PMC522873 15345050

[9] Kim HJ, Cui X, Hilsenbeck SG, Lee AV (2006) Progesterone receptor loss correlates with human epidermal growth factor receptor 2 overexpression in estrogen receptor-positive breast cancer. Clin Cancer Res 12: 1013s–1018s. 16467118 10.1158/1078-0432.CCR-05-2128

[10] Habashy HO, Powe DG, Staka CM, Rakha EA, Ball G, et al.. (2010) Transferrin receptor (CD71) is a marker of poor prognosis in breast cancer and can predict response to tamoxifen. Breast Cancer Res Treat. 10.1007/s10549-009-0345-x 19238537

[11] Gasparri F, Wang N, Skog S, Galvani A, Eriksson S (2009) Thymidine kinase 1 expression defines an activated G1 state of the cell cycle as revealed with site-specific antibodies and ArrayScan(TM) assays. European Journal of Cell Biology 88: 779–785. 19726104 10.1016/j.ejcb.2009.06.005

[12] Habashy HO, Rakha EA, Ellis IO, Powe DG (2013) The oestrogen receptor coactivator CARM1 has an oncogenic effect and is associated with poor prognosis in breast cancer. Breast Cancer Res Treat 140: 307–316. 23887673 10.1007/s10549-013-2614-y

[13] Habashy HO, Powe DG, Rakha EA, Ball G, Macmillan RD, et al (2010) The prognostic significance of PELP1 expression in invasive breast cancer with emphasis on the ER-positive luminal-like subtype. Breast Cancer Res Treat 120: 603–612. 19495959 10.1007/s10549-009-0419-9

[14] Popov VM, Zhou J, Shirley LA, Quong J, Yeow WS, et al (2009) The cell fate determination factor DACH1 is expressed in estrogen receptor-alpha-positive breast cancer and represses estrogen receptor-alpha signaling. Cancer Res 69: 5752–5760. 19605405 10.1158/0008-5472.CAN-08-3992 PMC3244171

[15] Cruz JA, Wishart DS (2006) Applications of Machine Learning in Cancer Prediction and Prognosis. Cancer Informatics 2: 59–77. PMC2675494 19458758

[16] McCulloch W, Pitts W (1943) A logical calculus of the ideas immanent in nervous activity. Bulletin of Mathematical Biology 5: 115–133. 2185863

[17] Lancashire LJ, Roberts DL, Dive C, Renehan AG (2011) The development of composite circulating biomarker models for use in anticancer drug clinical development. Int J Cancer 128: 1843–1851. 20549702 10.1002/ijc.25513

[18] Khan J, Wei JS, Ringner M, Saal LH, Ladanyi M, et al (2001) Classification and diagnostic prediction of cancers using gene expression profiling and artificial neural networks. Nature Medicine 7: 673–679. 10.1038/89044 PMC1282521 11385503

[19] Abd El-Rehim DM, Ball G, Pinder SE, Rakha E, Paish C, et al (2005) High-throughput protein expression analysis using tissue microarray technology of a large well-characterised series identifies biologically distinct classes of breast cancer confirming recent cDNA expression analyses. Int J Cancer 116: 340–350. 15818618 10.1002/ijc.21004

[20] Lisboa PJ, Taktak AF (2006) The use of artificial neural networks in decision support in cancer: a systematic review. Neural Netw 19: 408–415. 16483741 10.1016/j.neunet.2005.10.007

[21] Lancashire LJ, Rees RC, Ball GR (2008) Identification of gene transcript signatures predictive for estrogen receptor and lymph node status using a stepwise forward selection artificial neural network modelling approach. Artif Intell Med 43: 99–111. 18420392 10.1016/j.artmed.2008.03.001

[22] Dhondalay GK, Tong DL, Ball GR (2011) Estrogen receptor status prediction for breast cancer using artificial neural network; 2011 10-13 July 2011. pp. 727–731.

[23] Heanue TA, Davis RJ, Rowitch DH, Kispert A, McMahon AP, et al (2002) Dach1, a vertebrate homologue of Drosophila dachshund, is expressed in the developing eye and ear of both chick and mouse and is regulated independently of Pax and Eya genes. Mechanisms of Development 111: 75–87. 11804780 10.1016/s0925-4773(01)00611-6

[24] Mardon G, Solomon NM, Rubin GM (1994) Dachshund encodes a nuclear protein required for normal eye and leg development in Drosophila. Development 120: 3473–3486. 7821215 10.1242/dev.120.12.3473

[25] Wu K, Katiyar S, Witkiewicz A, Li A, McCue P, et al (2009) The cell fate determination factor dachshund inhibits androgen receptor signaling and prostate cancer cellular growth. Cancer Research 59: 3347–3355. 10.1158/0008-5472.CAN-08-3821 PMC2669850 19351840

[26] Wu K, Li A, Rao M, Liu M, Dailey V, et al (2006) DACH1 is a cell fate determination factor that inhibits cyclin D1 and breast tumor growth. Mol Cell Biol 26: 7116–7129. 16980615 10.1128/MCB.00268-06 PMC1592900

[27] Wu K, Jiao X, Li Z, Katiyar S, Casimiro MC, et al (2011) Cell fate determination factor Dachshund reprograms breast cancer stem cell function. J Biol Chem 286: 2132–2142. 20937839 10.1074/jbc.M110.148395 PMC3023510

[28] Wu K, Katiyar S, Li A, Liu M, Ju X, et al (2008) Dachshund inhibits oncogene-induced breast cancer cellular migration and invasion through suppression of interleukin-8. Proc Natl Acad Sci U S A 105: 6924–6929. 18467491 10.1073/pnas.0802085105 PMC2374551

[29] Shi L, Reid LH, Jones WD, Shippy R, Warrington JA, et al (2006) The MicroArray Quality Control (MAQC) project shows inter- and intraplatform reproducibility of gene expression measurements. Nat Biotechnol 24: 1151–1161. 16964229 10.1038/nbt1239 PMC3272078

[30] ArrayExpress website. Available: http://www.ebi.ac.uk/arrayexpress/. Accessed 2012 Mar 21.

[31] Lemetre C, Lancashire L, Rees R, Ball G (2009) Artificial Neural Network Based Algorithm for Biomolecular Interactions Modeling. In: Cabestany J, Sandoval F, Prieto A, Corchado J, editors. Bio-Inspired Systems: Computational and Ambient Intelligence: Springer Berlin Heidelberg. pp. 877–885.

[32] Shannon P, Markiel A, Ozier O, Baliga NS, Wang JT, et al (2002) Cytoscape: A Software Environment for Integrated Models of Biomolecular Interaction Networks. Genome Research 12: 2498–2504. 10.1101/gr.1239303 PMC403769 14597658

[33] Habashy HO, Powe DG, Rakha EA, Ball G, Paish C, et al (2008) Forkhead-box A1 (FOXA1) expression in breast cancer and its prognostic significance. Eur J Cancer 44: 1541–1551. 18538561 10.1016/j.ejca.2008.04.020

[34] McCarty KS Jr, Miller LS, Cox EB, Konrath J, McCarty KS Sr (1985) Estrogen receptor analyses. Correlation of biochemical and immunohistochemical methods using monoclonal antireceptor antibodies. Archives of Pathology and Laboratory Medicine 109: 716–721. 3893381

[35] Watanabe A, Ogiwara H, Ehata S, Mukasa A, Ishikawa S, et al (2011) Homozygously deleted gene DACH1 regulates tumor-initiating activity of glioma cells. PNAS 108: 12384–12389. 21750150 10.1073/pnas.0906930108 PMC3145721

[36] Nan F, Lu Q, Zhou J, Chen L, Popov VM, et al (2009) Altered expression of DACH1 and cyclin D1 in endometrial cancer. Cancer Biology and therapy 8: 1534–1539. 19502783 10.4161/cbt.8.16.8963

[37] Liang F, Lu Q, Sun S, Zhou J, Popov VM, et al (2012) Increased expression of dachshund homolog 1 in ovarian cancer as a predictor for poor outcome. Interanational Journal of Gynecolopgical Cancer 22: 386–393. 10.1097/IGC.0b013e31824311e6 22367319

[38] Wu K, Yang Y, Wang C, Davoli MA, D'Amico M, et al (2003) DACH1 inhibits transforming growth factor-beta signaling through binding Smad4. J Biol Chem 278: 51673–51684. 14525983 10.1074/jbc.M310021200

[39] DeAngelis T, Wu K, Pestell R, Baserga R (2011) The type 1 insulin-like growth factor receptor and resistance to DACH1. Cell Cycle 10: 1956–1959. 21558809 10.4161/cc.10.12.15800

[40] Ward S, Scope A, Rafia R, Pandor A, Harman S, et al.. (2012) Gene expression profiling and expanded immunohistochemistry tests to guide the use of adjuvant chemotherapy in breast cancer management. 10.3310/hta17440 PMC4780957 24088296

[41] Lancashire LJ, Powe DG, Reis JS, Rakha E, Lemetre C, et al (2010) A validated gene expression profile for detecting clinical outcome in breast cancer using artificial neural networks. Breast Cancer Res Treat 120: 83–93. 19347577 10.1007/s10549-009-0378-1

[42] Hoch RV, Thompson DA, Baker RJ, Weigel RJ (1999) GATA-3 is expressed in association with estrogen receptor in breast cancer. International Journal of Cancer 84: 122–128. 10096242 10.1002/(sici)1097-0215(19990420)84:2<122::aid-ijc5>3.0.co;2-s

[43] Fang SH, Chen Y, Weigel RJ (2009) GATA-3 as a marker of hormone response in breast cancer. J Surg Res 157: 290–295. 19059610 10.1016/j.jss.2008.07.015

[44] Wykoff CC, Beasley N, Watson PH, Campo L, Chia SK, et al (2001) Expression of the Hypoxia-Inducible and Tumor-Associated Carbonic Anhydrases in Ductal Carcinoma in Situ of the Breast. American Journal of Pathology 158: 1011–1019. 11238049 10.1016/S0002-9440(10)64048-5 PMC1850356

