# Supplementary figures and images for: DACH1: Its Role as a Classifier of Long Term Good Prognosis in Luminal Breast Cancer
Source: PLoS One. 2014 Jan 2;9(1):e84428. doi: 10.1371/journal.pone.0084428 (PMC3879319; doi:10.1371/journal.pone.0084428)

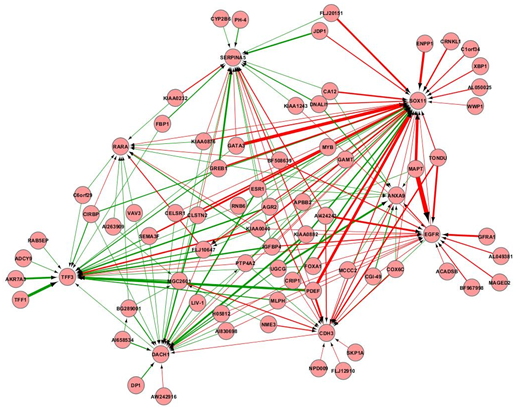

Supplement: Figure S1 — Interaction map of 2 (100 positive and 100 negative) interactions from highly predictive probe sets in ER positive samples. The genes are represented as nodes and interactions as edges. Green edge is a positive interaction and red edge is a negative interaction. The intensity of the interaction is represented in terms of the thickness of edge and the directionality with the arrow from source to target. The nodes with multiple interactions (>5) are considered as hubs. (TIF) [file pone.0084428.s001.tif]

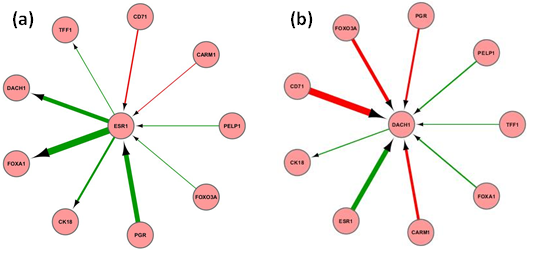

Supplement: Figure S2 — Association of luminal markers with (a) ESR1 and (b) DACH1 in luminal samples. The genes are represented as nodes and interactions as edges. The green edge is a positive interaction and the red edge is a negative interaction. The intensity of the interaction is represented in terms of the thickness of edge and the directionality with the arrow. (TIF) [file pone.0084428.s002.tif]
